# Sorting cells by their density

**DOI:** 10.1371/journal.pone.0180520

**Published:** 2017-07-19

**Authors:** Nazila Norouzi, Heran C. Bhakta, William H. Grover

**Affiliations:** Department of Bioengineering, University of California, Riverside, Riverside, CA, United States of America; Texas A&M University College Station, UNITED STATES

## Abstract

Sorting cells by their type is an important capability in biological research and medical diagnostics. However, most cell sorting techniques rely on labels or tags, which may have limited availability and specificity. Sorting different cell types by their different physical properties is an attractive alternative to labels because all cells intrinsically have these physical properties. But some physical properties, like cell size, vary significantly from cell to cell within a cell type; this makes it difficult to identify and sort cells based on their sizes alone. In this work we continuously sort different cells types by their density, a physical property with much lower cell-to-cell variation within a cell type (and therefore greater potential to discriminate different cell types) than other physical properties. We accomplish this using a 3D-printed microfluidic chip containing a horizontal flowing micron-scale density gradient. As cells flow through the chip, Earth’s gravity makes each cell move vertically to the point where the cell’s density matches the surrounding fluid’s density. When the horizontal channel then splits, cells with different densities are routed to different outlets. As a proof of concept, we use our density sorter chip to sort polymer microbeads by their material (polyethylene and polystyrene) and blood cells by their type (white blood cells and red blood cells). The chip enriches the fraction of white blood cells in a blood sample from 0.1% (in whole blood) to nearly 98% (in the output of the chip), a 1000x enrichment. Any researcher with access to a 3D printer can easily replicate our density sorter chip and use it in their own research using the design files provided as online Supporting Information. Additionally, researchers can simulate the performance of a density sorter chip in their own applications using the Python-based simulation software that accompanies this work. The simplicity, resolution, and throughput of this technique make it suitable for isolating even rare cell types in complex biological samples, in a wide variety of different research and clinical applications.

## Introduction

Biological and clinical samples are often heterogeneous populations of many different types of cells. Blood, for example, is a complex mixture of different cell types, only one of which may be needed for a given application. As a result, the ability to separate and sort cells by their type is fundamentally important in modern biological research and medical diagnostics.

Most existing cell sorting techniques can only be applied to certain types of cells. For example, fluorescence-activated cell sorting (FACS) and magnetically-activated cell sorting (MACS) rely on labels or tags that are intended to interact with certain cell types; these techniques are extremely powerful but cannot be used with cells that lack appropriate labels or tags. And even if, for example, an antibody specific to a particular cell type does exist, antibodies add significant cost to a procedure and complicate the translation of a sorting technique to clinical settings.

Sorting different cell types by their different physical properties is attractive because all cells intrinsically have these physical properties; no labels or tags are required. Consequently, cell sorters have been developed that sort cells based on physical properties like size [1], deformability [2], electrical polarizability [3], and others. However, for some physical properties, the intrinsic cell-to-cell variation of that property within a cell type can confound efforts to identify different cells by that property. For example, in human red blood cells (erythrocytes), the coefficient of variation in cell size is typically 11–15% [4]; while this variation (called *red cell distribution width*) has interesting clinical applications [5], it complicates attempts to distinguish red blood cells from other cells based on their size alone.

Of all the physical properties of cells that could be used to distinguish and separate cells of interest, *cell density*—the mass-to-volume ratio of a cell—is perhaps the most powerful. The intrinsic cell-to-cell variability of density is generally much lower than that of mass or volume: the coefficient of variation of the density of red blood cells is only about 0.5% [6], a factor of 20 smaller than the variation in cell size. As a result, in some cases cell types that are indistinguishable by size (mass or volume) *can* be distinguished by their density. For example, mouse leukemia cells undergo an increase in density mere minutes after treatment with a drug that induces apoptosis; this density increase is so significant that individual cancer cells can be identified as reacting to the drug based solely on their density, even though the mass and volume of the cells remain virtually unchanged [6].

The conventional tool for separating different cell types by their densities is the *density gradient*. In this almost-eighty-year-old technique, cells are placed in a fluid that contains a range of densities (more dense at the bottom, less dense at the top). The cells then sink or float to the location where the cell’s density is equal to the surrounding fluid’s density (this is the the cell’s *isopycnic point*, the location in the density gradient where the cell is neutrally buoyant). Bands containing cells of interest can then be retrieved from the gradient manually using a pipet.

While density gradients are powerful tools, a number of disadvantages severely limit their utility. First, the buoyant forces that move a cell to its isopycnic point in a density gradient are inherently weak. For a 20 *μ*m diameter spherical cell with a density that is 1% greater than the surrounding fluid density, the Earth’s gravitational acceleration (1 g) will pull the cell to its isopycnic point at a velocity of only 2 *μ*m/s or 7 mm/h. This cell would take several hours to travel to its isopycnic point in a centimeter-sized density gradient. In practice, this process can be accelerated by placing the gradient in a centrifuge and applying hundreds or thousands of g’s of acceleration (this is the basis for *density gradient centrifugation*). However, prolonged exposure to high g-forces can damage cells by *e.g*. forcing subcellular components out of the cells [7], and prolonged exposure to the concentrated solutions used to construct the density gradient can affect cell viability [8, 9]. Additionally, the resolution of the technique is limited by the quality and uniformity of the density gradient [10]. Constructing a smooth gradient of fluid densities that spans several centimeters is challenging, and inconsistencies in the density gradient lead to poor quality cell separation. Also, in density gradient centrifugation, dynamic range and resolution are mutually exclusive. A gradient that spans a wide range of densities can separate a wide range of cells by their densities but cannot discriminate small differences in cell densities; conversely, a gradient that spans a small range of densities can discriminate cells with small density differences but only over a narrow range of densities. Cell-to-cell interactions can also complicate density gradient separations: if two cells of different densities adhere together, they will sink or float to a location that represents an average of their two densities [11]. Manually retrieving cells of interest from the gradient is a laborious process that can affect the purity of the selected fraction of cells [7]. Moreover, as a “batch” technique, the throughput of density gradient centrifugation is limited to the capacity of a centrifuge tube. Finally, the cost and size of ultracentrifuges used for density gradient centrifugation also limit the utility of the technique in resource-limited settings.

**Fig 1:**
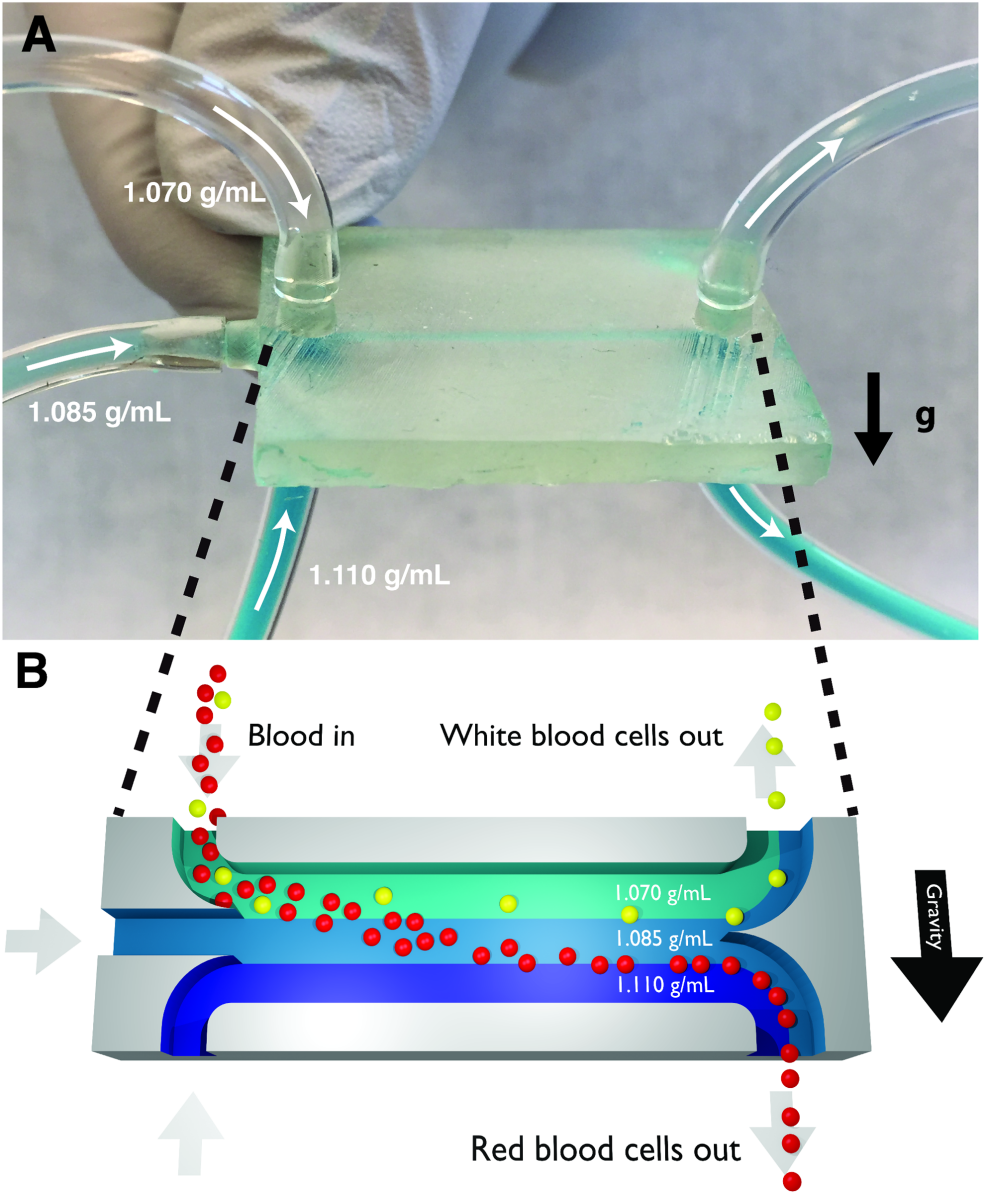
Photograph (A) and cross-section illustration (B) of a 3D-printed density sorter chip. Fluids with three different densities (1.070, 1.085, and 1.110 g/mL) are pumped into the inlets; under laminar flow conditions these fluids form a micron-scale density gradient flowing along the horizontal channel. If a mixture of blood cells is included in the top fluid, the flowing white blood cells (average density *ρ* = 1.080 g/mL) quickly sink to the interface between the 1.070 and 1.085 g/mL fluids where they are neutrally buoyant, and the flowing red blood cells (average density *ρ* = 1.110 g/mL) sink to the interface between the 1.085 and 1.110 g/mL fluids. When the channel splits, the white blood cells flow out of the top outlet and the red blood cells flow out of the bottom outlet.

In this work, we demonstrate a simple 3D-printed microfluidic chip that is capable of continuously sorting cells (and other samples) by their densities, without using a centrifuge or other significant hardware. The chip shown in Fig 1 contains several fluids with different densities. When these fluids flow together under laminar flow conditions, the fluids form what is effectively a micron-scale, continuously flowing density gradient. In this miniaturized density gradient, cells have a very short distance to travel before they reach their isopycnic point, and they travel this micron-scale distance in just a few seconds under the Earth’s 1 g of gravitational acceleration (no centrifuge is needed). When the channel then splits, cells with different densities collect in different outlets, ready for enumeration or further analysis. This technique can continuously sort a stream of cells by their densities alone (without being influenced by the cells’ other physical properties) and can isolate cell of interest from other cells with higher throughput and gentler conditions than existing tools.

In previous work, we showed that when two fluids of different densities flow together horizontally in a microfluidic chip, the fluids quickly reorient themselves relative to gravity (locating the more-dense fluid on the bottom and the less-dense fluid on the top) and form two stable flowing fluid layers of different densities [12]. In this work we show that any number of different-density fluids can be combined in this manner to create a continuously-flowing micron-scale on-chip density gradient capable of sorting cells by their densities. For example, in Fig 1, three streams of fluid with three different densities merge together into a single horizontal microfluidic channel. The most-dense fluid (1.110 g/mL) flows along the bottom of the channel; the middle-density fluid (1.085 g/mL) flows along the middle of the channel, and the least-dense fluid (1.070 g/mL) flows along the top of the channel. If one fluid also contains a mixture of cells, then the cells will not only travel horizontally through the channel with the fluid flow but also be move vertically to their isopycnic points due to buoyant forces. For example, if blood cells are added to the least-dense fluid, white blood cells with a density of 1.080 g/mL sink until they reach the interface between the 1.070 and 1.085 g/mL fluids, and red blood cells with a density of 1.110 g/mL sink until they reach the interface between the 1.085 and 1.110 g/mL fluids. When the channel then splits into two outlet channels, the different cell types are sorted to different destinations based on the cells’ densities.

**Fig 2:**
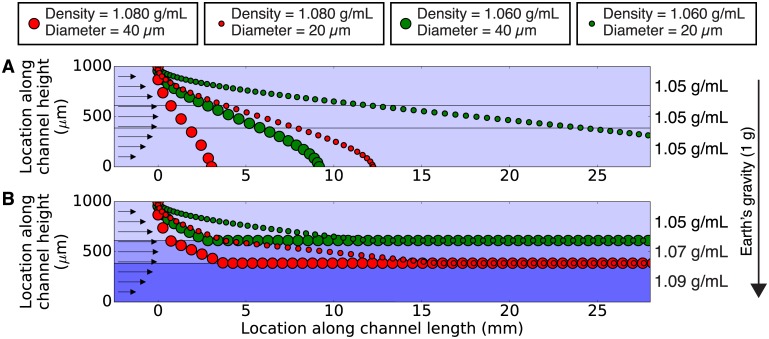
A simulation of paths followed by four different types of particles in a microchannel containing fluid with a uniform density (A; 1.05 g/mL) and in a microchannel containing three zones with different fluid densities (B; 1.05, 1.07, and 1.09 g/mL), obtained using our custom software density_sorter.py (S1 and S2 Files). In both cases, marker color connotes particle density (1.080 g/mL for red; 1.060 g/mL for green), and marker size connotes particle size (40 *μ*m diameter for the larger marker; 20 *μ*m diameter for the smaller marker). The particles all start at the same location at the top of the channel, and the position of each particle is calculated using Eqs 1 and 2 at 5 second intervals. Each fluid has a flow rate of 1.2 *μ*L/min, for a combined flow rate of 3.6 *μ*L/min. In a fluid of uniform density (**A**), the particles sink at different speeds but they all end up collecting on the bottom of the channel. However, in a continuously-flowing density gradient (**B**), both sizes of green (1.060 g/mL) particles sink until they reach the interface between the top two fluids, and both sizes of red (1.080 g/mL) particles sink until they reach the interface between the bottom two fluids. The particles then exit the channel in different locations and are thereby sorted according to their density alone (not their size).

To show why our chip is capable of sorting cells by their densities alone (and is not influenced by cell size), we calculated the trajectories followed by cells of different sizes and densities as they flow through the chip. Fig 2 shows the trajectories followed by four different types of objects flowing in two channels containing different fluid density profiles. The objects are representative of cells with different sizes (20 or 40 *μ*m diameters) and densities (1.060 or 1.080 g/mL). For simplicity, this is a two-dimensional simulation that represents the behavior of particles in a slice along the center of the microfluidic channel, and it models the channel as the gap between two horizontal parallel plates. Since the channel contents have a parabolic flow profile (ranging from zero flow at the channel walls to a maximum flow at the center of the channel), the velocity of an object in the *x*-direction *v*_*x*_ (that is, the object’s velocity in the direction of fluid flow) is determined solely by the object’s location in the *y*-direction (its vertical position in the channel):

$$\begin{array}{c} v_{x}=v_{xmax}\frac{4y(h-y)}{h^{2}} \end{array}$$ (1)

where *v*_*xmax*_ is the maximum flow velocity in the *x* direction, *h* is the height of the channel, and *y* is the *y*-coordinate of the location of the object in the channel. Additionally, the velocity of an object in the *y*-direction *v*_*y*_ (that is, the velocity at which the object floats up or sinks down in the direction of gravity) can be determined by Stokes’ Law:

$$\begin{array}{c} v_{y}=\frac{2}{9}\frac{\rho_{o}-\rho_{f}}{\mu_{f}}gr_{o}^{2} \end{array}$$ (2)

where *ρ*_*o*_ is the density of the object, *ρ*_*f*_ is the density of the fluid, *μ*_*f*_ is the viscosity of the fluid, *g* is the gravitational acceleration (−9.8 m/s^2^) and *r*_*o*_ the radius of the object. By calculating *v*_*x*_ and *v*_*y*_ over time and plotting *y* vs. *x* as shown in Fig 2, the paths followed by different cells in the chip can be predicted.

In a microfluidic channel containing fluid with a single uniform density (Fig 2A), cells introduced at the top of the channel follow trajectories that are a function of the cells’ densities *and* sizes [13–15]. For the combination of cell sizes and densities shown, the large and more-dense cells (large red circles) fall quickly and reach the channel floor first. They are then joined by the large and less-dense cells (large green circles), then the small and more-dense cells (small red circles) also reach the channel floor. The small and less-dense cells (small green circles) move the slowest in the *y* direction and reach the channel floor last, though not within the 25 mm shown. Thus, using fluid of a single uniform density, these four cells cannot be sorted by their densities alone. Instead, the cells’ locations and velocities are a function of both their size and their density, and the cells keep moving until they all eventually accumulate on the channel floor.

However, in a microfluidic channel containing fluid with *three different densities* (Fig 2B), the four types of cells introduced at the top of the channel reach locations that are *exclusively* a function of their densities (not their sizes). The large and more-dense cells (large red circles) sink until they reach the interface between the bottom two fluid densities; at this point the cells are neutrally buoyant and no longer move in the *y* direction but continue flowing in the *x* direction. The small and more-dense cells (small red circles) take more time to reach the same interface, but once they do, they (and all other cells with the same density) follow the same line as they flow through the channel. Similarly, both sizes of less-dense cells (large and small green circles) converge to the interface between the top two fluid densities and then stop moving in the *y* direction but continue flowing in the *x* direction. If the channel in Fig 2B were to then split in two, the top half would contain only the cells with density = 1.06 (green) and the bottom half would contain only the cells with density = 1.08 g/mL (red). In this manner, by using a microfluidic channel containing layers of fluids of different densities, cells (and other samples) can be continuously sorted by their densities alone.

## Results

### Sorting microbeads by polymer type

**Fig 3:**
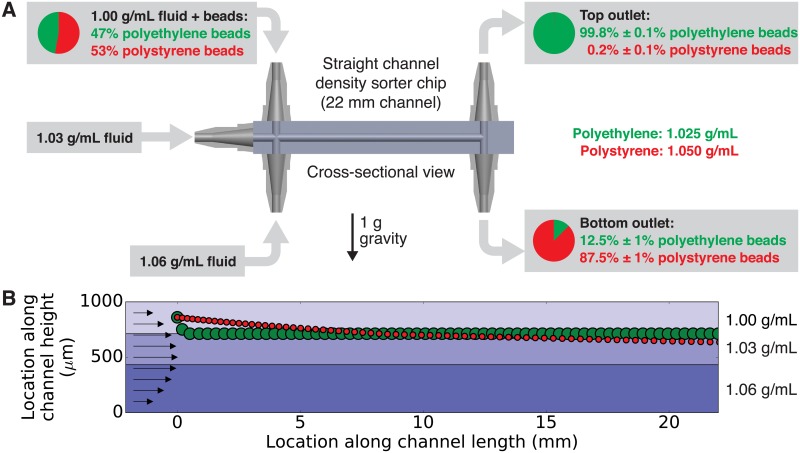
(**A**) Using the density sorter chip to sort microbeads by their density. The inlets of the density sorter chip received fluid of three different densities (1.00 g/mL in the top inlet, 1.03 g/mL in the middle inlet, and 1.06 g/mL in the bottom inlet). The top inlet fluid also contained a 47:53 mixture by number of polyethylene beads (density 1.025 g/mL) and polystyrene beads (density 1.050 g/mL). The beads collected at the top outlet were an average of 99.8% polyethylene, and the beads collected at the bottom outlet were an average of 87.5% polystyrene. The uncertainties in percentage points represent the standard deviation of three replicates of this experiment. (**B**) Using our density_sorter.py software (S1 File) to simulate this experiment and calculate the locations of the polyethylene and polystyrene beads at 5 second intervals. For a larger but less-dense polyethylene bead (green circle) starting in the middle of the top density layer, the bead reaches the interface between the top two density layers in around ten seconds and remains there as it flows through the chip. However, for a smaller but more-dense polystyrene bead starting in the same location, the bead does not quite reach the midpoint of the channel within the 22 mm channel length. This may explain the lower purity of polystyrene beads in the bottom outlet of the chip.

To validate our method, we used a 3D-printed microfluidic chip to sort microbeads made of materials with different densities. The design of the chip is available for download in standard STL format (S3 File). The experimental setup and results are summarized in Fig 3A. A mixture of polyethylene beads (40 *μ*m diameter; density = 1.025 g/mL) and polystyrene beads (6 *μ*m diameter; density = 1.050 g/mL) was prepared in a physiological buffer (density 1.00 g/mL) and pumped into the top inlet of the chip. The middle inlet received buffer with a density of 1.03 g/mL, and the bottom inlet received buffer with a density of 1.06 g/mL. Small amounts of Percoll (an inert and nontoxic colloidal silica solution) were used to increase the densities of these buffers without significantly affecting their other properties. Since Percoll-based density gradients are stable for months [16], we expect that the layers of different-density fluids in the chip remain well defined as they pass through the chip. The fluid densities of these layers were chosen to sort the less-dense polyethylene beads to the interface between the top two fluids and sort the more-dense polystyrene beads to the interface between the bottom two fluids. Fluid from each outlet was collected and analyzed using a hemocytometer. Of the beads collected from the top outlet, 99.8% were polyethylene and only 0.2% were polystyrene. Conversely, of the beads collected from the bottom outlet, only 12.5% were polyethylene and 87.5% were polystyrene. This demonstrates that the chip can successfully sort beads with only a small (2%) difference in bead density with separation efficiencies as high as 99%.

We also simulated this bead-sorting experiment using our software density_sorter.py (S1 File). The simulation results (Fig 3B) predict that the larger but less-dense polyethylene bead sinks to the interface between the 1.00 and 1.03 g/mL fluid densities very rapidly (in about 10 seconds). However, the smaller but more-dense polystyrene bead sinks more slowly and does not reach the midpoint of the channel before the channel splits at 22 mm. These results may explain the lower purity of the polystyrene beads at the bottom outlet, and they suggest that additional channel length may be needed to provide enough on-chip time for all polystyrene beads to reach the interface between the 1.03 and 1.06 g/mL fluids and thus exit the chip through the bottom outlet.

### Exploring the role of multiple fluid density layers

**Fig 4:**
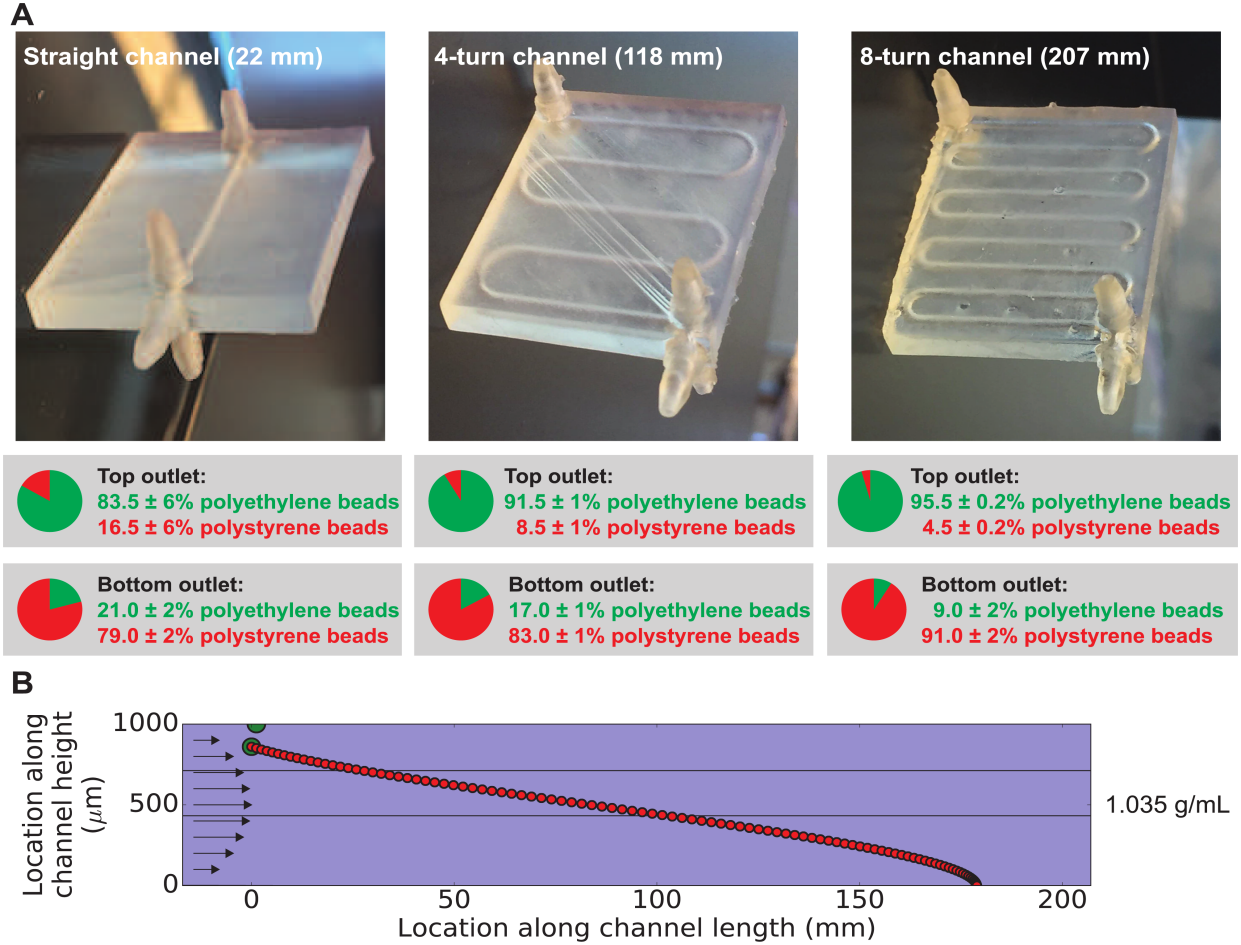
(**A**) To explore the role of fluid densities and channel lengths the operation of the density sorter chip, the bead-sorting experiment in Fig 3 was repeated using a single fluid density in all three inlets (1.035 g/mL) and three different channel lengths (22, 118, and 207 mm). Without layers of different fluid densities as in Fig 3, the purity of the beads at the outputs of the straight channel chip (22 mm) dropped from an average of 99.8% to 83.5% for the top outlet and from an average of 87.5% to 79.0% for the bottom outlet. Increasing the channel length increases the purity of the beads at each output, with an average of 95.5% polyethylene beads at the top outlet and an average of 91.0% polystyrene beads at the bottom outlet of the 8-turn (207 mm) density sorter chip. The uncertainties in percentage points represent the standard deviation of three replicates of each experiment. (**B**) For insights into these results, we simulated beads in the 207 mm chip using our density_sorter.py software (S1 File) at 30 second intervals. The larger but less-dense polyethylene bead (green circle) floats to the channel ceiling in about 30 seconds, and the smaller but more-dense polystyrene bead (red circle) sinks to the channel floor in about 3000 seconds. These results suggest that without multiple density layers, particles are free to sink or float to the channel walls where they may stick irretrievably (thus reducing sorting efficiencies compared to the results in Fig 3), though lengthening the channel can improve sorting efficiencies somewhat.

A key difference between our technique and previous work [14, 15] is our use of multiple fluid densities in our density sorter chip, effectively creating a continuously-flowing micron-scale density gradient in the horizontal channel. To determine whether our technique outperforms existing sorter chips that use a single fluid density, we repeated the microbead sorting experiment in Fig 3 using a single fluid density (1.035 g/mL) throughout the chip. This fluid density was chosen because it is roughly halfway between the densities of the polyethylene and polystyrene beads, so the polyethylene beads should float to the top outlet and the polystyrene beads should sink to the bottom outlet. The results from the straight channel (22 mm) in Fig 4A show that when only one fluid density is used, the average purity of the beads at the outlets decreases, dropping from 99.8% to 83.5% polyethylene beads for the top outlet and from 87.5% to 79.0% polystyrene beads for the bottom outlet. The decrease in purity is most pronounced at the top outlet. To understand why, we used our density_sorter.py software to simulate this experiment. The results (Fig 4B) show that the larger and less-dense polyethylene bead floats relatively quickly and reaches the top of the channel after flowing only about 1 mm along the horizontal channel. For the remaining 21 mm of the channel, the polyethylene bead is essentially rolling along the ceiling of the channel. This provides opportunities for the bead to become stuck in the channel, effectively reducing the number of of polyethylene beads that exit the chip and decreasing the purity of the polyethylene beads at the top outlet. In contrast, when using multiple fluid densities (Fig 3B), fluid densities can be chosen that eliminate bead-to-wall contact and result in higher separation efficiencies.

### Exploring the role of channel length

In principle, giving a bead or other object more time to sink or float to its isopycnic point should improve the separation efficiency of our technique. One way to accomplish this would be to decrease the flow rate through the density sorter chip, but this would have the disadvantage of decreasing the throughput of our technique. A better way to accomplish this would be to lengthen the channel containing the flowing density gradient, so that the cells flow at the same speed but spend more time in the micron-scale density gradient before the channel splits. To test whether a longer channel would actually improve our separation efficiency, we designed and 3D printed additional chips with serpentine geometries shown in Fig 4A. While our original straight-channel chip has a 22 mm long channel containing the flowing density gradient, the four-turn chip has a 118 mm long channel and the eight-turn chip has a 207 mm long channel. To focus exclusively on the effect of channel length, we pumped fluid of a uniform density (1.035 g/mL) into all three inlets, with the top inlet also containing a 47:53 mixture of the same polyethylene and polystyrene beads used above. As expected, additional channel length does improve separation efficiency somewhat: lengthening the channel from 22 to 207 mm increased the fraction of polyethylene beads leaving the top outlet from 83.5% to 95.5% and increased the fraction of polystyrene beads leaving the bottom outlet from 79.0% to 91.0%.

These improvements in separation efficiency with increased channel length are fairly modest. To understand why, we used our density_sorter.py software to simulate the behavior of beads in the 8-turn channel (207 mm) chip. The simulation results (Fig 4B) show that, as mentioned earlier, in fluid of uniform density the larger and less-dense polyethylene bead floats to the ceiling of the channel after only 1 mm of travel along the channel. Additionally, the smaller and more-dense polystyrene bead sinks to the floor of the channel after about 180 mm of travel along the channel. The resulting interactions between the beads and the channel walls can lead to stuck beads and decreased sorting efficiency. These results suggest that using multiple fluid densities (as in Fig 3) is a more powerful strategy than lengthening the horizontal channel (as in Fig 4) for increasing the separation efficiency of our technique.

### Sorting blood cells by type

**Fig 5:**
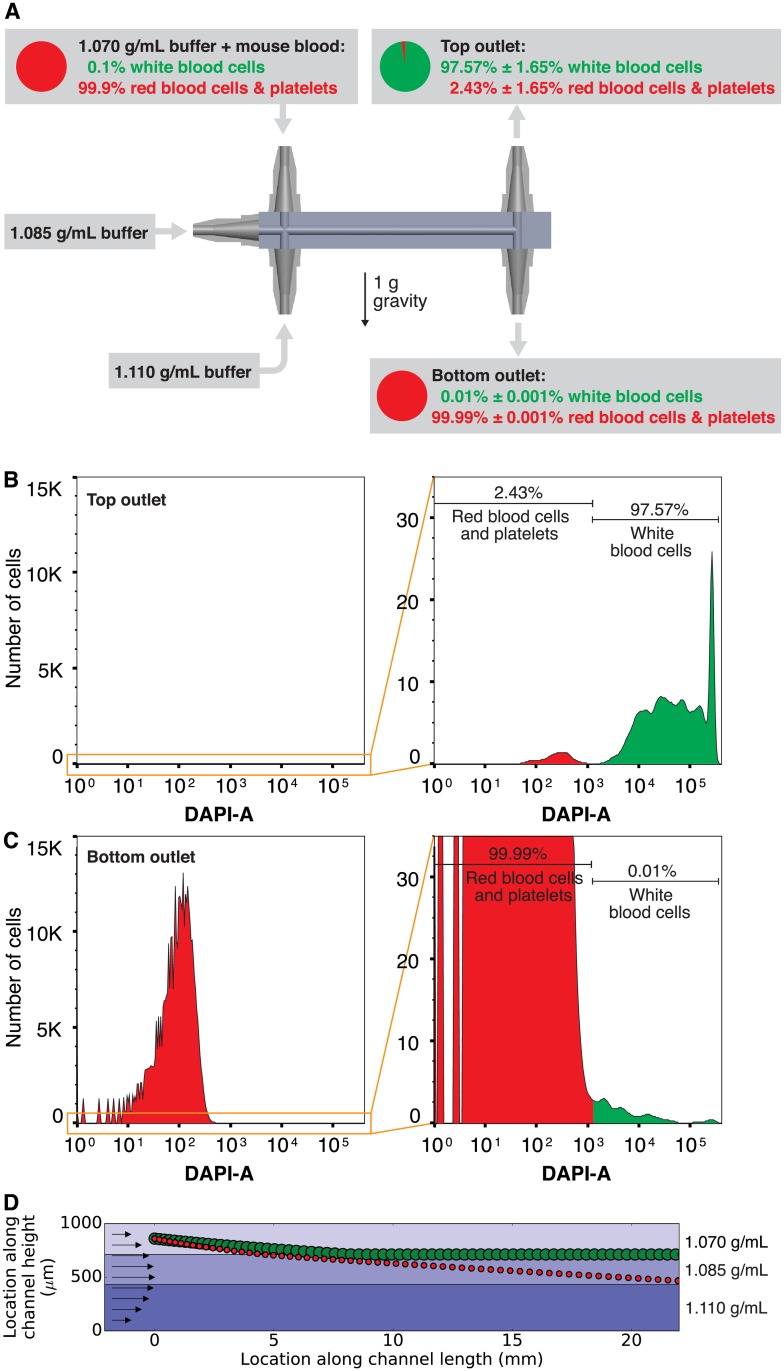
Using the density sorter chip to sort white blood cells from red blood cells and platelets. (**A**) Mouse blood (which normally contains 0.1% white blood cells) was diluted in a 1.070 g/mL buffer and pumped into the top inlet; the middle inlet received 1.085 g/mL buffer and the bottom inlet received 1.110 g/mL buffer. Cells collected at the outlets were then stained using DAPI to distinguish white blood cells from other cells. Flow cytometry analysis of the stained cells indicates an average of 97.57% of the cells exiting the top outlet are white blood cells (**B**; a nearly 1000× enrichment), and an average of 99.99% of the cells exiting the bottom outlet are red blood cells and platelets (**C**). The uncertainties in percentage points represent the standard deviation of three replicates of this experiment; cell counts for each replicate are shown in Table 1. By simulating this experiment using our density_sorter.py software at 5 second intervals (**D**), we see that a typical white blood cell (large green circle) reaches the interface between the top two fluid densities at 155 seconds and remains there, but a typical red blood cell (small red circle) starting at the same location barely reaches the middle of the chip in the 22 mm channel length. This could explain the presence of 2.4% red blood cells in the top outlet and suggests that lengthening the channel could further improve separation efficiency.

To demonstrate sorting cells by type using their different densities, we used the same 3D-printed microfluidic chip from Fig 3A to sort blood into two fractions: one containing white blood cells, and another containing the other cells (red blood cells and platelets). As shown in Fig 5A, buffers with three different densities were pumped into the three inlets of the chip (1.070 g/mL in the top inlet, 1.085 g/mL in the middle inlet, and 1.110 g/mL in the bottom inlet). These fluid densities were chosen to sort the white blood cells (density approximately 1.08 g/mL) to the interface between the top two fluids, and sort the red blood cells (density approximately 1.11 g/mL) to the interface between the bottom two fluids. The top buffer also included a 1-in-500 dilution of whole mouse blood; this dilution ratio was chosen to provide a cell concentration at the outputs of the density sorter chip that is suitable for analysis by flow cytometry. Cells from each outlet were collected, stained with DAPI (4′,6-diamidino-2-phenylindole; a fluorescent stain that binds to the DNA that is present in white blood cells but absent from red blood cells and platelets), and analyzed using a fluorescence-activated cell sorter in Fig 5B and 5C. Even though white blood cells are relatively rare in mouse blood (only 0.1% of cells [17]), an average of 97.57% of the cells from the top outlet were white blood cells, a nearly 1000× enrichment. The bottom outlet contained only 0.01% white blood cells, or about a fifth of the white blood cells present in whole blood. This experiment was performed in triplicate with similar results each time (actual cell counts for each replicate are shown in Table 1).

**Table 1:** Counts of DAPI+ cells (white blood cells) and DAPI− cells (red blood cells and platelets) from three replicates of the blood cell sorting experiment in Fig 5.

	Top outlet		Bottom outlet	
Replicate	DAPI+	DAPI−	DAPI+	DAPI−
1	5764	42	193	7 × 10^6^
2	5599	145	238	6 × 10^6^
3	5280	222	107	6 × 10^6^

The enrichment of white blood cells at the top outlet of the chip is significant, but a nontrivial amount of red blood cells and platelets (average 2.43%) contaminate the cells in the top outlet. To understand why these cells are here, we used our density_sorter.py software to simulate the behavior of red blood cells and white blood cells in this experiment. The results (Fig 5D) show that while the white blood cells sink to the interface between the 1.070 and 1.085 g/mL fluids after just 8 mm of flow through the channel, the red blood cells barely make it past the midpoint of the channel after flowing through the full 22 mm length of the channel. Thus, some red blood cells may not have enough time in the channel to sink to their isopycnic point, and these cells may exit the top outlet as contaminants. Increasing the channel length (while still using three fluid densities) would likely increase the purity of the white blood cells at the top outlet.

Finally, for some cell sorting applications, it is important to demonstrate not only the purity of the sorted cells but also the throughput of the sorter. One microliter of mouse blood has approximately 6000 white blood cells and 9 × 10^6^ red blood cells and platelets [17]. When this sample of blood is sorted using the density sorter chip, an average of 5700 white blood cells and 6.3 × 10^6^ red blood cells and platelets are collected (Table 1) were collected from the outlets of the chip. Therefore, about 95% of the red blood cells and 70% of the white blood cells that enter the chip are collected at the outputs. Some of the lost cells likely remain in the tubing connected to the inlets and outlets of the density sorter chip and could be retrieved by rinsing the chip at the end of a sorting operation.

## Conclusion

The density-based cell sorter presented here offers several advantages over existing techniques like density gradient centrifugation. First, by using a miniaturized density gradient, our chip does not need hundreds or thousands of g’s of acceleration to separate cells by their density. Instead, Earth’s 1 g is adequate to separate samples with small (2%) differences in density in just a few seconds in the density sorter chip. Consequently, our technique eliminates the threat of g-force-induced damage to cells and also significantly reduces the cost of our technique and the amount of instrumentation required to perform it. Additionally, our chip uses simple laminar flow to automatically form the desired density gradient on-chip, a significant improvement over centrifugation techniques that require careful manual construction of the gradient. And by using chips with additional inlets supplied with fluids of different densities, continuously-flowing density gradients can be created with any desired density range and resolution. As a continuous-flow technique, our cell sorter eliminates virtually all of the manual labor associated with transferring samples into and collecting fractions out of density gradients. Finally, since our density sorter chips were fabricated using 3D printing, anyone with access to a 3D printer can download our design files and use our chips in their own research.

What are the limitations of this technique? One important limitation is particle size: there is a minimum size below which a particle’s random travel by diffusion will be faster than its vertical travel by buoyancy or sedimentation, so our method would not be able to sort the particle by its density. The distance *d* a particle will travel by diffusion in time *t* can be approximated by

$$\begin{array}{c} d=\sqrt{Dt} \end{array}$$ (3)

where *D* is the diffusion constant of the particle. We can approximate *D* for a particle using the Stokes-Einstein equation,

$$\begin{array}{c} D=\frac{k_{B}T}{6\pi \eta r} \end{array}$$ (4)

where *k*_*B*_ is Boltzmann’s constant, *T* is the absolute temperature, *η* is the dynamic viscosity of the fluid surrounding the particle, and *r* is the radius of the (assumed spherical) particle. Solving Eq 4 for a 9 *μ*m diameter red blood cell in water at room temperature, we obtain *D* = 2.4 × 10^−14^ m^2^/s. Substituting this value into Eq 3, we predict that during the 500 seconds that a typical red blood cell spends in the microfluidic density chip in a sorting experiment like the one shown in Fig 5, the cell may move in a random direction by about 3.6 *μ*m due to diffusion. This distance is much smaller than the distance the cell will travel vertically in the same time due to sedimentation (Eq 2), so we conclude that diffusion does not negatively affect the performance of the density sorter chip for cell-sized objects.

So how small would an object need to be for diffusion to outweigh sedimentation in the density sorter chip? If we assume that diffusing vertically a distance of 100 *μ*m would be far enough for a particle to exit its intended density layer and enter another density layer in the chip, and the particle still spends 500 seconds in the chip, the particle would need to have a diffusion constant of *D* = 1.89 × 10^−11^ m^2^/s to diffuse about 100 *μ*m. Using the Stokes-Einstein equation, this would correspond to a particle size of about 12 nm. This represents our estimate of the smallest particle that could *possibly* be sorted by its density using the density sorter chip.

The purity of the cells sorted by our chip (like the 99.99% pure erythrocytes/platelets and the 97.57% pure leukocytes isolated from whole blood in Fig 5) is already high enough for many analyses. For applications requiring even higher purity, our results in Fig 4 show that lengthening the horizontal channel increases our separation efficiency without affecting the throughput of our technique. And for applications requiring higher throughput (for example, to isolate extremely rare cells of interest like circulating tumor cells), a density sorter chip containing hundreds of parallel horizontal channels could be easily designed and fabricated using 3D printing. Indeed, by leveraging 3D printing in the fabrication of these chips, we are not limited to two-dimensional chips and can create three-dimensional density sorter “cubes” for extremely high throughput: a 3D-printed cube 15 cm on each side could contain 10,000 parallel density sorter channels and have 10,000× higher throughput than the chips presented here. In this manner, our density sorter is uniquely customizable for applications requiring high purity, high throughput, or both.

## Materials and methods

### Density sorter chip design and fabrication

Density sorter chips like the ones shown in Figs 1 and 4 were designed using SolidWorks (Dassault Systèmes, Vélizy-Villacoublay, France), exported as an STL file (S3 File), and printed using a stereolithography-based 3D printer (Form 1+, Formlabs, Cambridge, MA). The chips contain microfluidic channels with circular cross sections (1 mm diameter) and varying lengths (a straight 22 mm long channel was used in the experiments in Figs 3A, 4A (left), and 5A; and 118 and 207 mm long serpentine channels were used in the remaining experiments in Fig 4A).

### Density sorter chip simulation

The simulations in Figs 2A, 2B, 3B, 4B, and 5D were performed using density_sorter.py, a custom Python program available for download (S1 and S2 Files). This software solves Eqs 1 and 2 at user-specified time intervals, for particles with specified densities and sizes, starting at user-specified locations, in a channel containing any desired combination of fluid density layers. For example, this Python code was used to generate the blood cell sorting simulation shown in Fig 5D:

import density_sorter as ds
chip = ds.Chip(channel_height = 1e-3, channel_depth = 1e-3, channel_length = 0.022)
red_blood_cells = ds.Particle(radius = 9e-6/2.0, density = 1110.0)
white_blood_cells = ds.Particle(radius = 13.5e-6/2.0, density = 1080.0)
bottom_fluid = ds.Fluid(density = 1110.0, flow_rate = 1.66e-11)
middle_fluid = ds.Fluid(density = 1085.0, flow_rate = 1.66e-11)
top_fluid = ds.Fluid(density = 1070.0, flow_rate = 8.33e-12)
ds.simulate(chip, fluids = (bottom_fluid, middle_fluid, top_fluid), particles = (white_blood_cells, red_blood_cells), delta_t = 5)

This code defines a density sorter chip containing a 1 mm high, 1 mm deep, and 22 mm long channel. It then defines two types of particles, red_blood_cells (9 *μ*m diameter and 1.11 g/mL density) and white_blood_cells (13.5 *μ*m diameter and 1.08 g/mL density). It also defines three types of fluids, bottom_fluid (1.110 g/mL, flowing into the chip at 1.0 *μ*L/min), middle_fluid (1.085 g/mL, flowing into the chip at 1.0 *μ*L/min), and top_fluid (1.070 g/mL, flowing into the chip at 0.5 *μ*L/min). Finally, the code simulates the behavior of red_blood_cells and white_blood_cells in the chip containing bottom_fluid, middle_fluid, and top_fluid, at five second time intervals. The results are shown in Fig 5D. Complete code for generating Figs 2A, 2B, 3B, 4B, and 5D are available for download (S1 and S2 Files).

### Operating the density sorter chip

Fluids can be pumped into and out of the density sorter chip using several different techniques. In the experiments in Figs 3, 4, and 5, syringe pumps were used to control the flow of fluid through the chips. The chips were printed with barbed tubing fittings at the three inlets and two outlets. Tygon tubing (Cole-Parmer, Vernon Hills, IL) with an inner diameter of 1.6 mm was used to connect the inlets and outlets to the syringe pumps.

### Sorting beads by their density

For the experiment in Fig 3, polyethylene microbeads (40 *μ*m diameter, 1.025 g/mL density) and polystyrene microbeads (6 *μ*m diameter, 1.05 g/mL density) were obtained from Cospheric (Santa Barbara, CA). The beads were suspended in a buffer containing 1× phosphate buffered saline (PBS) and 0.05% Tween-20; final solution density 1.00 g/mL. To create the different-density fluids for the middle and bottom inlets, Percoll (GE Life Sciences, Piscataway, NJ) was added to the buffers. Percoll (a non-toxic aqueous solution of 15–30 nm colloidal silica coated with polyvinylpyrrolidone) has a relatively high density (1.13 g/mL) and can be used to adjust the density of a solution without significantly affecting the solution’s ionic strength or other properties. In Fig 3A, 5 mL of Percoll were combined with 16.67 mL of 1× PBS (to create the 1.03 g/mL fluid for the middle inlet) or 5.83 mL of 1× PBS (to create the 1.06 g/mL fluid for the bottom inlet). All three fluids were loaded into syringe pumps that were connected to the inlets of the 3D-printed chip using tubing. The buffer containing the beads was pumped into the top inlet using a flow rate of 0.5 *μ*L/min, and the other two fluids were pumped into the middle and bottom inlets using a flow rate of 1.0 *μ*L/min for each. The outlets of the chip were connected to another syringe pump operating in withdrawing mode; it collected the fluid from the outlets at a rate of 1.25 *μ*L/min for each of the two outlets.

For the experiments in Fig 4, all three inlets received a 1× PBS/Percoll solution with a density of 1.035 g/mL, and the length of the horizontal channel in the density chip was varied from 22 mm (Fig 4A), 118 mm (Fig 4B), and 207 mm (Fig 4C). All other experimental conditions were the same as in Fig 3.

### Sorting blood cells by their density

Blood was drawn from a healthy mouse into a tube containing the anticoagulant ethylenediaminetetraacetic acid (EDTA). This research was approved by the UC Riverside Institutional Animal Care and Use Committee under protocol number A-20140010. 1 *μ*L of whole blood was diluted in 500 *μ*L of 1x PBS buffer containing 4.28 mL of Percoll; final solution density 1.070 g/mL. PBS and Percoll solutions with densities of 1.085 g/mL and 1.110 g/mL were also prepared as described above. These fluids were pumped into and out of the density sorter chip using the same experimental conditions used for the bead experiments.

## Supporting information

S1 File Python code for simulating the behavior of density sorter chips. Used to create Figs 2, 3B, 4B, and 5D.
(ZIP)

S2 File A user guide for the density sorter chip simulation software. (PDF)

S3 File The design of the 3D-printed density sorter chip in standard STL format. Used to print the density sorter chips used in Figs 3, 4A, and 5.
(STL)
