# Supplementary figures and images for: Sorting cells by their density
Source: PLoS One. 2017 Jul 19;12(7):e0180520. doi: 10.1371/journal.pone.0180520 (PMC5516969; doi:10.1371/journal.pone.0180520)

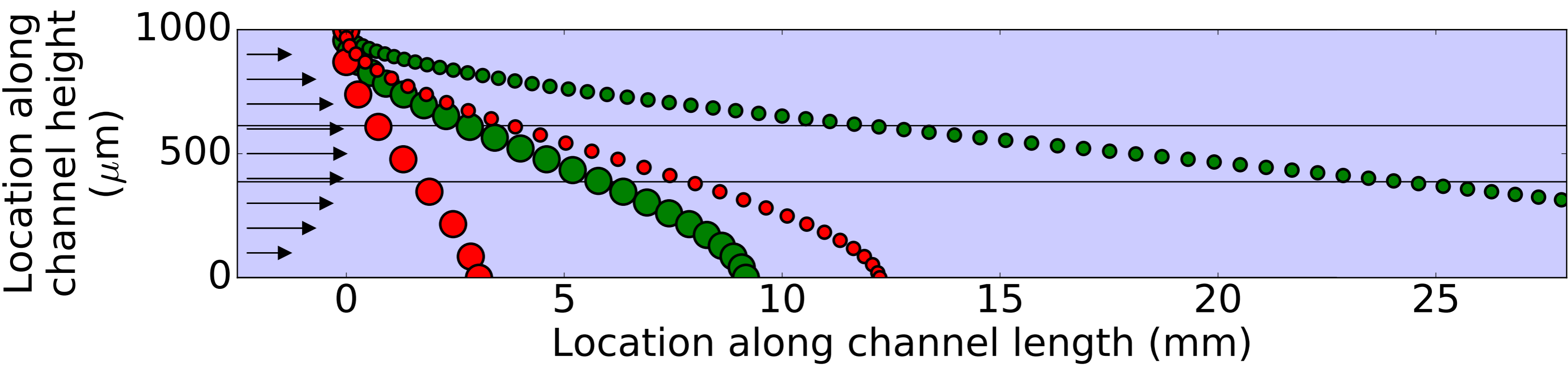

Supplement: S1 File — Used to create Figs 2, 3B, 4B, and 5D. (ZIP) [file pone.0180520.s001.zip › fig2a.pdf]

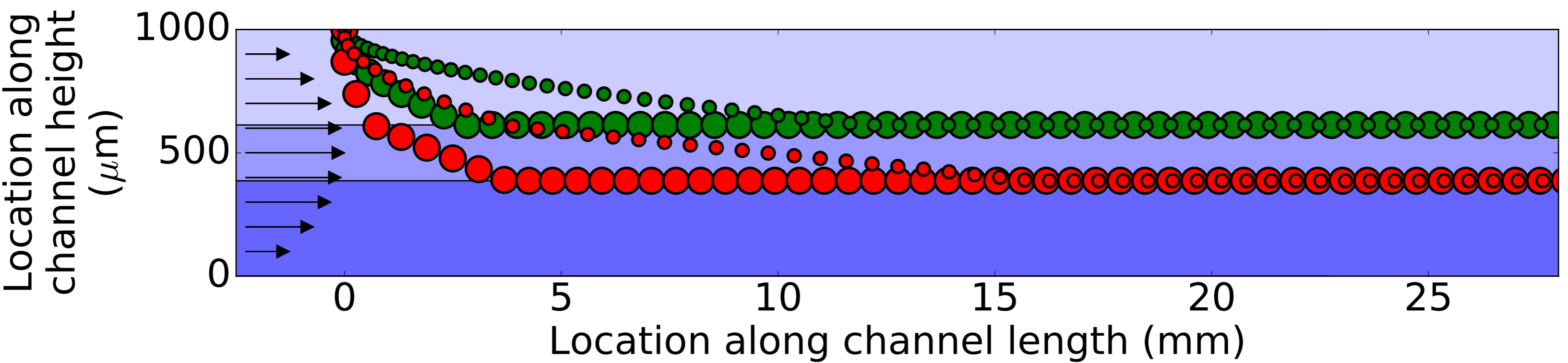

Supplement: S1 File — Used to create Figs 2, 3B, 4B, and 5D. (ZIP) [file pone.0180520.s001.zip › fig2b.pdf]

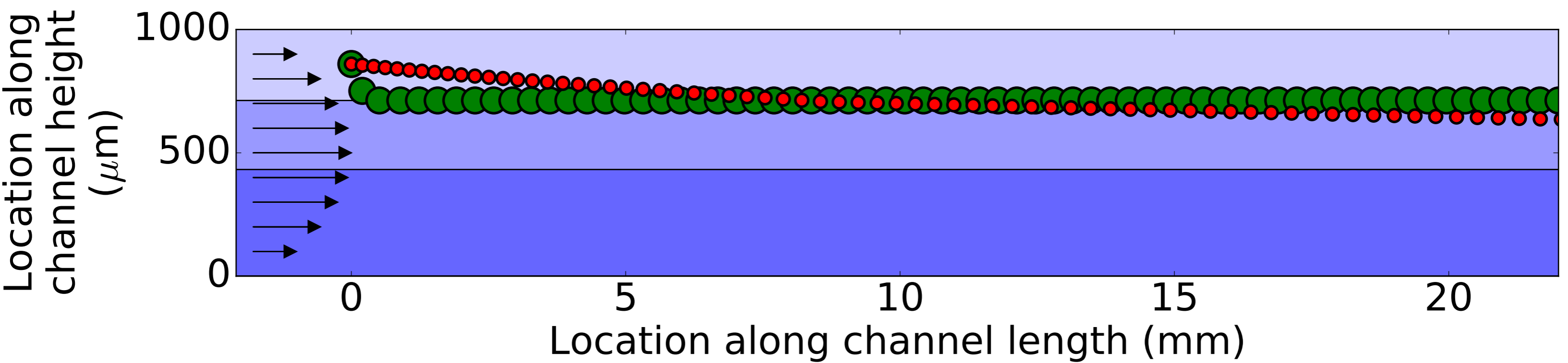

Supplement: S1 File — Used to create Figs 2, 3B, 4B, and 5D. (ZIP) [file pone.0180520.s001.zip › fig3b.pdf]

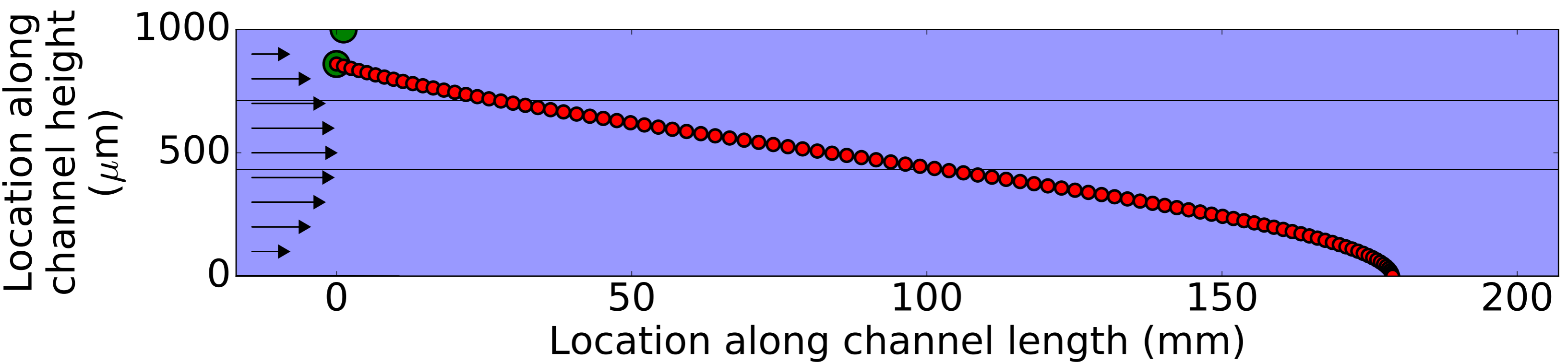

Supplement: S1 File — Used to create Figs 2, 3B, 4B, and 5D. (ZIP) [file pone.0180520.s001.zip › fig4b.pdf]

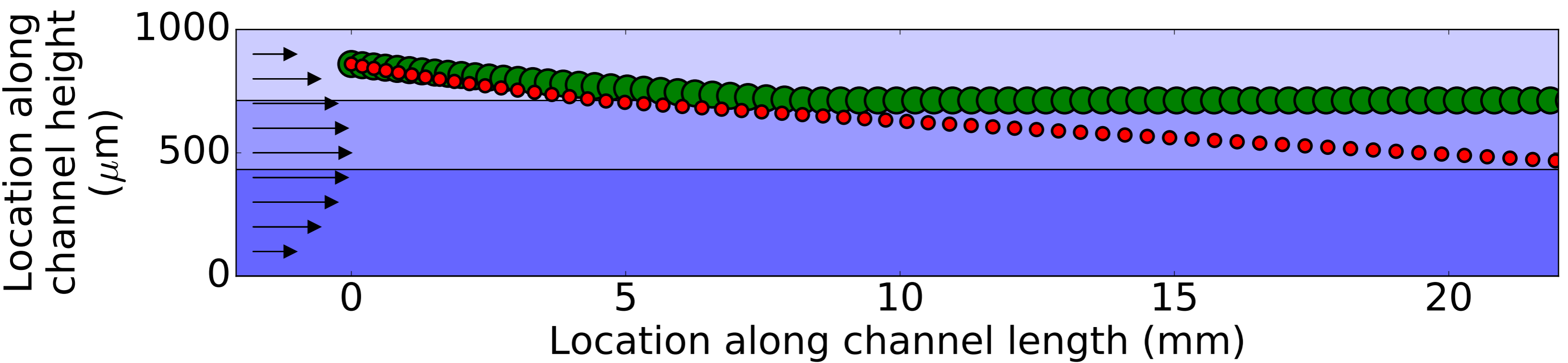

Supplement: S1 File — Used to create Figs 2, 3B, 4B, and 5D. (ZIP) [file pone.0180520.s001.zip › fig5d.pdf]
