# Supplementary material for: Sorting cells by their density
Source: PLoS One. 2017 Jul 19;12(7):e0180520. doi: 10.1371/journal.pone.0180520 (PMC5516969; doi:10.1371/journal.pone.0180520)
Supplement: S2 File — (PDF) [file pone.0180520.s002.pdf]

Using the density sorter chip simulation software density_sorter.py

as described in "Sorting cells by their density" by
Nazila Norouzi, Heran C. Bhakta, and William H. Grover
PLOS ONE 10.1371/journal.pone.0180520
Department of Bioengineering, University of California, Riverside

A custom Python program, `density_sorter.py`, was written to simulate the behavior of particles in density sorter chips. The current version of the software is available as online Supplementary Information, and the latest version of the software is available for download from `https://groverlab.org`. The software depends on three additional free Python packages that must be installed first: *numpy* (`http://numpy.org`), *scipy* (`http://scipy.org`), and *matplotlib* (`http://matplotlib.org`). These dependencies can be downloaded and installed individually, or they also come bundled with science-focused free Python distributions like *Anaconda* (`http://www.continuum.io`).

Importing the density_sorter.py package

To use `density_sorter.py`, first create an empty Python file in the same directory as `density_sorter.py`. In the blank file, add the following line to import the `density_sorter.py` package:

```
import density_sorter as ds
```

Next, add lines that define the density sorter chip, the particles to be sorted in the chip, and the fluid densities in the chip.

Specifying the density sorter chip

The density sorter chip is created as an instance of the `ds.Chip` object. For example, this code,

```
chip = ds.Chip(channel_height=1e-3, channel_depth=1e-3,
               channel_length=0.022)
```

creates a density sorter chip called `chip` which contains a channel with a height of 1×10^{-3} m (1 mm), a depth of 1×10^{-3} m (1 mm), and a length of 0.022 m (22 mm). Channel height is in the dimension parallel to Earth's gravity (*i.e.*, the distance from the floor to the ceiling of the channel). Channel depth is the width of the channel perpendicular to gravity. Channel length is the distance from the beginning of the horizontal channel (where two or more fluids with different densities merge together) to the end of the channel (where the fluids split into two or more outlets).

Specifying the particles to be sorted

Particles are instances of the `ds.Particle` object. For example, this code,

```
red_blood_cells = ds.Particle(radius=9e-6/2.0, density=1110.0,
                              location=(0, chip.channel_height*0.86),
                              marker_size=10, marker_color="r")
```

creates a particle called `red_blood_cells` with a radius of 4.5×10^{-6} m (4.5 μm) and a density of 1110.0 kg/m^3 (1.110 g/mL). The starting location of the particle in the channel is $x = 0$ (meaning that the particle originates at the point along the channel where the two or more fluids of different densities merge together) and $y =$ `chip.channel_height` $\times$ 0.86 (meaning that the particle originates at a point that is 86% of the way to the top or ceiling of the channel; this represents a location that is in the middle of the topmost fluid density layer in this experiment). The remaining arguments for the particle, `marker_size=10` and `marker_color="r"`, specify the size (10 units) and color (red) to use when plotting the trajectory of this particle in the simulation results. Refer to the documentation for the Python plotting package *matplotlib* for details on these arguments.

For a second example of a particle object, here is the code that creates another particle, this one called `white_blood_cells`:

```
white_blood_cells = ds.Particle(radius=13.5e-6/2.0, density=1080.0,
                                location=(0, chip.channel_height*0.86),
                                marker_size=20, marker_color="g")
```

This particle has a different radius and density but starts in the same location as the `red_blood_cells` particle. It will be plotted using a larger marker colored green.

Specifying the fluids in the chip

Fluids are instances of the `ds.Fluid` object. For example, this code,

```
f1 = ds.Fluid(density=1110.0, viscosity=0.001, flow_rate=1.66e-11,
              color='#6666ff')
```

```
f2 = ds.Fluid(density=1085.0, viscosity=0.001, flow_rate=1.66e-11,
              color='#9999ff')
f3 = ds.Fluid(density=1070.0, viscosity=0.001, flow_rate=8.33e-12,
              color='#ccccff')
```

creates three different fluids. The first one, named `f1`, has a density of 1110.0 kg/m^3 (1.110 g/mL), a viscosity of 0.001 kg m^{-1} s^{-1}, and a flow rate into the chip of 1.66×10^{-11} m^3/s (1.00 μL/min). Note that in the current version of `density_sorter.py`, the viscosity of the fluid is used when calculating the velocity of a particle in that fluid (solving Equation 2 in the main text), but not when calculating the horizontal flow profile in the channel (Equation 1 in the main text). Therefore, for accurate results, currently the viscosity of all fluids in a simulation should be equal. Finally, the argument `color='#6666ff'` specifies the color of the fluid to use when plotting the simulation results; this color is the hexadecimal representation of a dark blue color. Similarly, fluid `f2` has a unique density (1.085 g/mL) and color (medium blue), and `f3` has a unique density (1.070 g/mL) and color (light blue).

Running the simulation

Once the chip, the particles to be sorted, and the fluids inside the chip are defined, the simulation is run using the `ds.simulate()` function. For example, the code

```
ds.simulate(chip, fluids=(f1, f2, f3),
            particles=(white_blood_cells, red_blood_cells), delta_t=5,
            yticks=[0, 500, 1000], xlim=[-2.1, 22])
```

runs a simulation of the chip named `chip` containing the fluids `f1`, `f2`, and `f3`. The fluids are specified in the order in which the flow into the chip, from lowest (closest to Earth) to highest. To form a conventional density-gradient-like stack of densities in the horizontal channel (with the most-dense fluid on the bottom and the least-dense fluid on the top), this means that the most-dense fluid should be specified first (`f1` in this example) and the least-dense fluid should be specified last (`f3`). Note that if fluids are pumped into a chip in an order that places a more-dense fluid *above* a less-dense fluid, the layers may automatically reorient themselves to place the less-dense layer on the bottom [1]. The `density_sorter.py` software supports any number of different fluids with different densities. The argument `particles=(white_blood_cells, red_blood_cells)` specifies which particles to simulate in the chip; again, any number of different particle types are supported. The argument `delta_t=5` specifies the time resolution of the simulation; in this example, Equations 1 and 2 from the main text will be solved for each particle at five second increments, and a marker specifying the location of each particle will be plotted every five seconds. Finally, the argument `yticks=[0, 500, 1000]` specifies where to place tick marks on the y axis of the simulation results (in units of μm), and the argument `xlim=[-2.1, 22]` specifies the range of the x axis of the simulation results (the length of the plot in the direction of the channel length, in units of mm); the documentation for the Python plotting package *matplotlib* has

more details on these arguments.

Obtaining the simulation results

Combining the preceding lines of code into a single file creates the code needed to generate Fig 5d in the main text:

```
import density_sorter as ds
chip = ds.Chip(channel_height=1e-3, channel_depth=1e-3,
               channel_length=0.022)
red_blood_cells = ds.Particle(radius=9e-6/2.0, density=1110.0,
                              location=(0, chip.channel_height*0.86),
                              marker_size=10, marker_color="r")
white_blood_cells = ds.Particle(radius=13.5e-6/2.0, density=1080.0,
                                location=(0, chip.channel_height*0.86),
                                marker_size=20, marker_color="g")
f1 = ds.Fluid(density=1110.0, viscosity=0.001, flow_rate=1.66e-11,
              color='#6666ff')
f2 = ds.Fluid(density=1085.0, viscosity=0.001, flow_rate=1.66e-11,
              color='#9999ff')
f3 = ds.Fluid(density=1070.0, viscosity=0.001, flow_rate=8.33e-12,
              color='#ccccff')
ds.simulate(chip, fluids=(f1, f2, f3),
            particles=(white_blood_cells, red_blood_cells), delta_t=5,
            yticks=[0, 500, 1000], xlim=[-2.1, 22])
```

This code generates the following plot, also shown as Fig 5D in the main text:

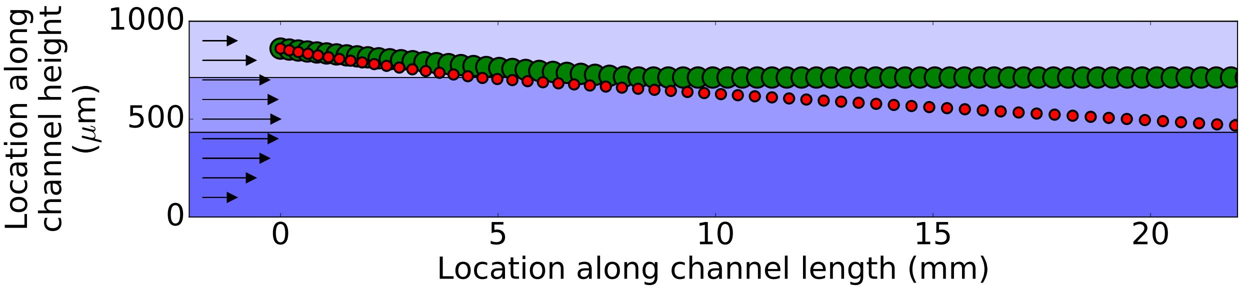

The Python file for this and all other simulations shown in the manuscript are available for download as online *Supplementary Information*:

- `fig2a.py`: Code for generating the simulation in Fig 2A
- `fig2b.py`: Code for generating the simulation in Fig 2B
- `fig3b.py`: Code for generating the simulation in Fig 3B

- `fig4b.py`: Code for generating the simulation in Fig 4B
- `fig5d.py`: Code for generating the simulation in Fig 5D

References

[1] Nazila Norouzi, Heran C Bhakta, and William H Grover. Orientation-based control of microfluidics. *PLOS ONE*, 11(3):e0149259, 2016.
